# US–Japan Cooperative Medical Sciences Program’s Virtual Workshop on COVID-19

**DOI:** 10.3201/eid2710.211779

**Published:** 2021-10

**Authors:** Yohei Doi, Ken Ishii, Akira Nishizono, Asuka Nanbo, Shuzo Urata, Ichiro Kurane, Jules Carl Celebrado, John Philip Depatillo, Zymar Bandola, Diane E. Griffin, Eun-Chung Park, David McDonald, Kristina Lu, K. Gayle Bernabe, Gray Handley

**Affiliations:** Fujita Health University School of Medicine, Toyoake, Japan (Y. Doi);; University of Pittsburgh School of Medicine, Pittsburgh, Pennsylvania, USA (Y. Doi);; University of Tokyo, Tokyo, Japan (K. Ishii);; Oita University Faculty of Medicine, Oita, Japan (A. Nishizono);; National Research Center for the Control and Prevention of Infectious Diseases, Nagasaki University, Nagasaki, Japan (A. Nanbo, S. Urata);; National Institute of Infectious Diseases, Tokyo (I. Kurane);; Department of Science and Technology, Manila, Philippines (J.C. Celebrado, J.P. Depatillo, Z. Bandola);; Johns Hopkins Bloomberg School of Public Health, Baltimore, Maryland, USA (D.E. Griffin);; National Institute of Allergy and Infectious Diseases, National Institutes of Health, Bethesda, Maryland, USA (E.-C. Park, D. McDonald, K. Lu, K.G. Bernabe, G. Handley)

**Keywords:** COVID-19, coronavirus disease, SARS-CoV-2, severe acute respiratory syndrome coronavirus 2, viruses, respiratory infections, zoonoses, United States, Japan, US–Japan Cooperative Medical Sciences Program, USJCMSP, International Conferences on Emerging Infectious Diseases

The US–Japan Cooperative Medical Sciences Program, a 56-year-old bilateral program, has convened the International Conferences on Emerging Infectious Diseases in the Pacific Rim in different countries in the Asia–Pacific region since 1996 (*1*,*2*). Because of the ongoing pandemic, the annual conference could not convene in person. Rather, a virtual workshop on coronavirus disease (COVID-19) was held February 24–26, 2021, focusing on virology and immunology of the disease, as well as the intersection and impact on other infectious diseases, particularly in the region (Table).

**Table:** Workshop presenters at the International Conference on Emerging Infectious Diseases in the Pacific Rim, February 24–26, 2021

Date and theme	Workshop presenters
February 24 (day 1): Clinical Research and Epidemiology	Dr. Babatunde Olowokure (World Health Organization Health Emergencies Programme, Western Pacific Region Office, Philippines); Prof. Benjamin John Cowling (University of Hong Kong, Hong Kong SAR); Dr. Firdausi Qadri (Centre for Vaccine Sciences at icddr,b, Bangladesh); Dr. Andrew S. Azman (Johns Hopkins Bloomberg School of Public Health, USA); Prof. Marissa Alejandria (National Institutes of Health of the University of the Philippines Manila, Philippines); Prof. Akihide Ryo (Yokohama City University School of Medicine, Japan); and Dr. Florian Krammer (Icahn School of Medicine at Mount Sinai, USA)
February 25 (day 2): Vaccines, Immunology, Therapeutics, and Diagnostics	Prof. Ken Ishii (University of Tokyo, Japan); Dr. Nina Gloriani (College of Public Health, University of the Philippines Manila, Philippines); Dr. Mario Antonio Jiz (Research Institute for Tropical Medicine, Philippines); Prof. Yohei Doi (Fujita Health University School of Medicine, Japan); Dr. Nicole Basta (McGill University, Canada); and Dr. Yoshimasa Takahashi (National Institute of Infectious Diseases, Japan)
February 26 (day 3): Virology, Zoonosis, and One Health	Dr. Maria Rosario Vergeire (Department of Health, Philippines); Prof. Yoshihiro Kawaoka (University of Tokyo, Japan); Dr. Galit Alter (Harvard University, USA); Prof. Ling Fa Wang (Duke– National University of Singapore Medical School, Singapore); Dr. Peter Daszak (EcoHealth Alliance, USA); Dr. Frederic Béen (KWR Water Research Institute, Netherlands)

The workshop started with global and regional updates on COVID-19, including clinical and epidemiologic studies and sharing of experiences. The information included the World Health Organization’s approach to prevent and suppress the transmission of COVID-19 by exhaustive surveillance and delivery of safe and effective vaccines and therapeutics. For pandemic preparedness, lessons learned from pandemic influenza may be leveraged to guide future responses. The speaker emphasized the need for research and discovery of universal vaccines against pathogens from genetically diverse virus families. Regional updates included a report that an increase in reported respiratory infections among school children in Hong Kong was attributable to rhinovirus infections and not to severe acute respiratory syndrome coronavirus 2 (SARS-CoV-2), suggesting that transmission of these common cold viruses may be less affected by the mitigation approaches, and a report on the negative impact of the pandemic on cholera-focused activities in Bangladesh, where cholera vaccination efforts remain a priority and will be redoubled along with the roll-out of COVID-19 vaccines.

Diagnostics were addressed by several speakers. In a presentation that outlined the serologic characteristic of SARS-CoV-2 infection and the latest technologic advances aimed at increasing sensitivity and specificity of viral infection diagnostics, the speaker noted the importance of considering the disease prevalence and the influence of factors, such as age and disease severity, among different groups within a population in developing assay platforms, and a need for the development of SARS-CoV-2–specific IgA diagnostic tests. Another speaker presented seroepidemiologic data on SARS-CoV-2, highlighting the considerable variability that exists in the sensitivity and specificity among SARS-CoV-2 serologic assays on the market and the highly variable background cross-reactivity observed across populations. Serologic assays are also essential for epidemiologic and vaccine efficacy studies. A research team presented a quantitative ELISA and a novel neutralizing assay using virus-like particles that detected neutralizing antibodies in >80% of patient samples at 6 months after SARS-CoV-2 infection. In another project, plasma collected from infected persons was used to detect early signatures in naturally infected persons to track SARS-CoV-2 mortality and identify possible biomarkers to guide clinical practice and address surges of infection. Another presenter described a surrogate virus neutralization test based on antibody-mediated blockage of the ACE2-spike protein–protein interaction. The concept was to provide a simulation of live virus neutralization as a diagnostic format to detect the presence of antibodies blocking spike interaction with the ACE2 receptor and could be used for monitoring effective humoral immune responses to vaccination.

Discussion on therapeutics and vaccines was kicked off by an overview of the factors affecting SARS-CoV-2 infection outcomes and treatment strategies, including pathogenesis of COVID-19, demographic risk factors for severe disease and death, and the emergence of SARS-CoV-2 variants. The next presenter described the role of innate immune cells, specifically polymorphonuclear-myeloid-derived suppressor cells and SARS-CoV-2 receptor binding domain–specific IgG titers in severe COVID-19. Therapeutic antibodies targeting mutating sites, in which monoclonal antibodies with SARS-CoV-1 and -2 cross-neutralizing activities and resistance to escape variants have been isolated, suggest that cross-neutralizing antibodies are advantageous tools for treatment of COVID-19. For vaccines, work was presented on mRNA encoding the receptor binding domain of the viral spike protein encapsulated in lipid nanoparticles, and its efficacy was experimentally evaluated in mice and confirmed in cynomolgus monkeys infected with SARS-CoV-2, providing possibilities for additional mRNA vaccines. Another talk provided a summary of natural and vaccine-induced immune responses to SARS-CoV-2 infection and discussed how an ideal immune response to the vaccine should mimic the natural immune response to SARS-CoV-2. The next speaker introduced an online platform designed to track the changing landscape of COVID-19 vaccine candidates (https://covid19.trackvaccines.org). Goals for this website include improvement of public health communication to inform the public about vaccine status and contributions to epidemiologic and population health research. The platform tracks the progress of COVID-19 vaccine candidates, including clinical trial information. With regard to antivirals, clinical trials on the effect of favipiravir on COVID-19 were summarized, highlighting how positive results were observed during early time points of viral infection even though favipiravir treatment did not have a protective effect at later time points, likely a common theme for direct-acting antivirals.

The workshop was wrapped up by talks on zoonoses, One Health, and the experience with COVID-19 in the Philippines. Similar to other countries, the Philippines experienced immense challenges, but it has managed the pandemic by taking a multisectoral approach and by ensuring that decision-making is informed by science. A speaker presented research on animal models to address SARS-CoV-2 transmission dynamics. The efficiency of masks in inhibiting aerosol transmission was demonstrated by using a hamster model and showed that masks worn by the exhaling hamster are more efficient than masks worn by the receiving ones in preventing transmission. Other presentations addressed systems serologic analysis and an analysis of geographic hotspots of viral disease emergence, including the emergence of novel coronaviruses recorded in the last thousand years. Although Southern China is a hotspot, other areas in the region are also at high risk because humans are in close contact with highly diverse wildlife species. The final talk focused on opportunities and challenges with conducting environmental sewage surveillance of SARS-CoV-2 as part of the One Health approach. Wastewater surveillance may be used for early warning, determine unbiased trends, detect hotspots at a city level or in closed communities, and detect variants through next-generation sequencing.

The hope is that the workshop, which was organized by the US–Japan Cooperative Medical Sciences Program Scientific Planning Committee and the Philippines’ Department of Science and Technology, will further promote and foster continuing and new research cooperation through and beyond the pandemic. Additional information about the program and the conference is available at https://www.niaid.nih.gov/research/us-japan-cooperative-program-organization-history and https://www.amed.go.jp/news/event/210224_Conference.html.

## References

[1] Rao M, Hall R, Lu K, Mason R, Bernabe G, Brennan P, et al. 21st International Conference on Emerging Infectious Diseases in the Pacific Rim. Emerg Infect Dis. 2019;25.

[2] Lu KT, Yamamoto T, McDonald D, Li W, Tan M, Moi ML, et al. U.S.-Japan cooperative medical sciences program: 22nd International Conference on Emerging Infectious Diseases in the Pacific Rim. Virology. 2021;555:71–7. 10.1016/j.virol.2020.12.012 33454559 PMC7870554

